# The MISLI-Drive, a modular sterilizable robotic driver for steerable laparoscopic instruments

**DOI:** 10.3389/frobt.2023.1227708

**Published:** 2023-10-06

**Authors:** Tomas Lenssen, Radu Bîrjac, Jenny Dankelman, Tim Horeman

**Affiliations:** Minimally Invasive Surgery and Interventional Techniques (MISIT)-Lab, Department of Biomedical Engineering, Delft University of Technology, Delft, Netherlands

**Keywords:** instrument driver, laparoscopy, RAS, reusable, robotic surgery, sustainable

## Abstract

**Introduction:** Based on the success of the former “Shaft-Actuated, Tip-Articulated” SATA-Drive, a prototype robotic instrument driver for modular, steerable, laparoscopic instruments, a new driver is designed and tested to improve previously lacking features concerning cleanability, instrument adaptation, practical application and control. The design of the driver engages these issues with a modular design aimed at re-use of both the instrument and the driver, for which a set of design requirements are established.

**Methods:** A new modular design has been developed to improve cleanability through separation of the electro-motors and the instrument mechanism which clutches the instrument. Contamination of the driver’s robotic side is prevented though a combination of a drape and a Sterile barrier interface, while the instrument side is made sterilizable. A novel instrument clutching mechanism enables quick-release features, while a motor-axis latching mechanism enables plug-and-play assembly. Embedded sensors allow precise and fast control. A user-experiment was conducted on instrument exchange and assembly time, while mechanical and electrical tests were conducted on the driver’s responsiveness.

**Results:** The driver has proven its ability to control the instrument, after which it can be disassembled for cleaning and inspection. The driver is designed for re-use through disassembled sterilization where all possibly contaminated surfaces are exposable for cleaning and inspection. The new standardized instrument clutches allow easy instrument (dis-)assembly. Instrument exchange is possible in two methods, the fastest of which is a median of 11 (6.3–14.6) seconds. The driver’s instrument mechanism is separated in a median of 3.7 (1.8–8.1) seconds. After assembly, the driver is operational in less than 2 s.

**Discussion:** Instrument exchange times are similar to the semi-reusable Da Vinci systems, yet the MISLI-Drive is designed for sterilization, inspection and continual re-use. The modular build of the driver also allows easier parts replacement during maintenance, and requires minimal adaptation to different future scenarios, which is expected to reduce the overall cost of use.

## 1 Introduction

Robot Assisted Surgery (RAS) has improved the surgeon’s abilities in laparoscopic surgery in terms of stability, precision and control. Robotic instruments often have increased Degree of Freedom (DOFs) at their end-effector which allow for local steering at the surgical site (Idrees and Bartlett, 2010). However, this increase in instrument capacity has come with a coinciding increase in instrument complexity. In robot assisted laparoscopic systems, like the Da Vinci, most instruments use multiple sets of cables inside a non-accessible chassis which can only be flushed without further inspection, which makes the instrument limited-reusable. This has significantly increased purchasing costs and cleaning complications regarding residual contamination (Saito et al., 2017). General re-use of medical devices, as well as the ability to selectively purchase, combined with a focus on low production costs, should reduce the overall cost of robotic surgery, making it more accessible. Some instruments that tackle these issues do exist. The company “Asensus” has developed reusable and cableless instrument, but these are not steerable (Darwich and Stephan, 2022). Similarly, the company “Distalmotion” developed steerable instruments with a focus on sustainability through lowered complexity, but these are nonetheless sing-use (Digitalmotion, 2023).

**FIGURE 1:**
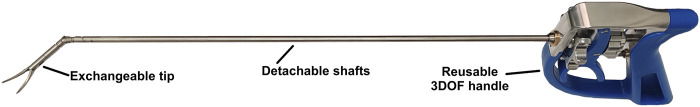
The hand-held SATA-LRS with double articulated end-effector.

**FIGURE 2:**
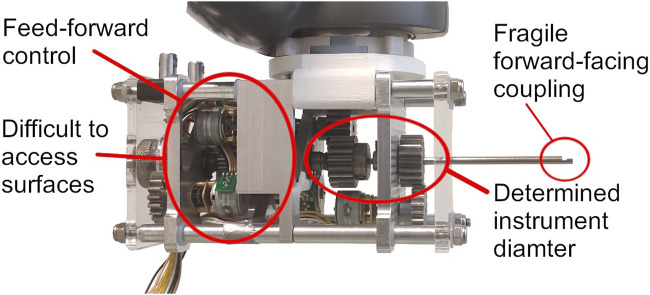
The SATA-Drive prototype with exposed coupling without instrument.

The SATA-LRS (see Figure 1) is a 2-DOF laparoscopic instrument with a cableless steering mechanism developed specifically for cleanability in low resource settings (Lenssen et al., 2022). Through a modular design, instrument disassembly allows cleaning, inspection and maintenance on a component level, which creates benefits in sterilization and purchasing costs. The SATA-Drive (see Figure 2), a prototype of a robotic driver for the SATA-LRS (Lenssen et al., 2023b), has proven the potency of this instrument in a robotic setting regarding ease of control, precision and instrument exchanges. The driver’s benefits in modularity, small footprint and instrument adaptation furthermore proved a promising design. However, this preliminary prototype did not meet the requirements relevant for the operating room or sterilization department. Analysis of the system showed several shortcoming that need to be addressed, which are also indicated in Figure 2.

Though the driver has no direct contact with the surgical site, the general hollow pipe structures of laparoscopic instruments do allow a constant flow of gas escaping from the abdomen into the instrument-driver. This presents a risk of contamination of the driver and consequently following instruments. Yet, the semi-permanent shafts and in-build electro-motors made it difficult to have the driver cleaned. The semi-permanent shafts also dedicated the driver’s build to a single instrument diameter, requiring a significant re-build to adopt other diameters. Though instrument exchange was intuitive and fast, the instrument’s disassembly required for decoupling needed a significant forward-facing space towards the patient, obligating the re-orientation of the robotic system to avoid collision with the surgical site. The so-called Puzzle-Piece-Connection (PPC), the coupling method used in the shafts of the instrument (Hardon et al., 2019), has also shown to be fragile in the smaller shaft diameters. Last, the SATA-Drive lacked any feedback systems and was relying entirely on a feed-forward control of its stepper motors. Hence, a new system, improving on these issues, was required.

This work describes the development of the MISLI-Drive, a Modular, Interfaced, Sterilizable Robotic Driver for Laparoscopic Instruments, particularly the SATA instrument-line, that can be easily cleaned and allows quick instrument exchanges without the risk of contamination or instrument damage. Instrument exchanges can be considered significant events during a robotic intervention (Hanly et al., 2004) and can happen as often as 15 times (Sgarbura and Vasilescu, 2010; Hanly et al., 2004). Sgarbura and Vasilescu (2010) found that locally trained surgical assistants for the Da Vinci (Intuitive surgical) require on average about 7.7 (3.7SD) seconds to exchange an instrument. However, two unforeseen cases required up to 1 and 7 min (Hanly et al., 2004; Sgarbura and Vasilescu, 2010). This indicates how an intuitive, fail-safe system can make an important difference in the total operation time. Therefore, the goal of this work is to develop a modular MISLI-Drive able to grasp multiple SATA instruments in a quick-release fashion.

## 2 Materials and methods

The requirements for the MISLI-Drive mentioned in the introduction are summarized in Table 1 according the SMART quantification method (Doran, 1921). The driver should make it possible to exchange instruments, previously possible within 30 s (Lenssen et al., 2023b), without robot re-orientation. Following the SATA-LRS’s model, the driver should be sterilizable, inspectable and exchangeable by a sterilisation department employee to make the system reusable, which is shown to be an overall cost-reducing feature in surgical equipment as well as an improvement in environmental factors (Rizan and Bhutta, 2022). The cleanability of the device will therefore be measured by the ability to directly access internal surfaces of the device. The total production cost of the driver will also be limited to 200€, similar to the SATA-Drive, by a focus on cost-effective production methods to improve procurement costs and accessibility to future robotic surgery. Last, embedded position feedback of the instrument will enable quick and precise control with a latency of less than 25 ms and a precision of 2°.

**TABLE 1:** Overview of all specific design requirements.

#	Design requirements	Motivation	Method	Goal
1	Instrument is detachable from driver	Exchange and cleanability	Quick-release system	All shafts and end-effector
2	MI is detachable from MU	Exchange and cleanability	Quick-release system	Remove IM from the sterile site
3	Adaptive instrument clutching	Use multiple instrument	Standardized clutch design	Shaft size of 3–5 mm
4	Low instrument exchange time	Support inter-operative exchanges	Quick-release system	15 s
5	No arm reorientation required	Support inter-operative exchanges	Backwards decoupling	Independent from arm
6	Instant instrument control	Support inter-operative exchanges	Orientation sensors and quick-latching system	2 s
7	Precise instrument control	Surgical applications	Position feedback and stepper-motors	0.5° accuracy and 50 ms latency
8	Focus on cost reduction	Affordable and accessible	Fully re-usable system	Production price of 200€
9	Reusable system through cleanability	Affordable and accessible	Detachable parts and casings	Internal surfaces exposable
10	Low weight	Handling and transport	Material choices	500 g or less

**FIGURE 3:**
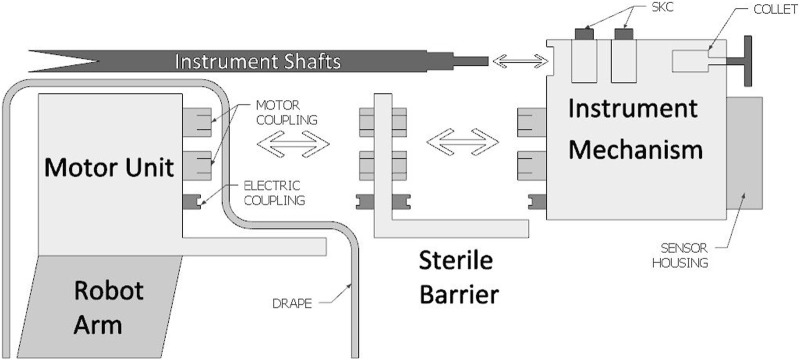
A schematic overview of the driver components separated into the 3 sub-systems: the Motor Unit (MU), Sterile Barrier Interface (SBI) and Instrument Mechanism (IM).

The focus of the design for the MISLI driver lies around a mechanism that holds the SATA instruments and can be easily detached with or without instrument. Since motor-electronics are difficult to clean and sterilize, the fundamental decision was made to split the driver in two: an Instrument Mechanism (IM) holding the instrument, and a Motor Unit (MU) housing the electro-motors (See Figure 3). Further aspects of the device have been developed according to the “bare-minimum design” approach, with a focus on component interaction and feature extension (Horeman et al., 2015; Hardon et al., 2019; Postema et al., 2021). To keep overall complexity low, multiple uses and factors are applied to the same part.

### 2.1 Driver assembly and contamination management

Figure 3 shows the MU designed to use a flange-side for semi-permanent attachment to the robot arm, which is typically covered by a drape. The IM, which stays uncovered by the drape, consists of a mostly mechanical system fit for cleaning and sterilization. To avoid direct contact between the sterile IM and the MU, a Sterile Barrier Interface (SBI) is used to transfer the required coupling between the two systems. Besides the motor coupling, all three sub-systems also contain an electric-coupling to transfer power and data from the MU to the IM. Before operation, the SBI is connected to the MU through a quick-release system involving so-called click-fingers. Before, or during, the operation the IM can then be connected to the SBI through a separate quick-release click-finger system, after which it can be removed or replaced at any time during or after the operation. The IM contains the shaft grasping mechanisms, which control the instrument, as well as the position feedback sensors. While instrument coupling is possible from the front of the mechanism, extraction of the IM is done backwards.

### 2.2 Instrument shaft decoupling

**FIGURE 4:**
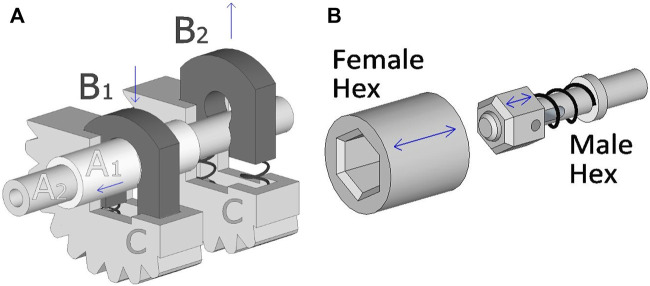
**(A)**: A schematic working principle of two Shaft-Key-Connection (SKC) each holding a shaft. (A_1*&*2_) a double instrument shaft, (B_1_) a key in the open position, (B_2_) a key in the locked position, and (C) a turnable chassis with a geared exterior. **(B)**: A schematic example of the Hex-Key (HK) self-latching systems.

The previous SATA-Driver using a Puzzle-Piece-Connection (PPC) effectively divided the instrument shaft in two sections of which one side was semi-permanently attached to the driver, complicating decontamination and inspection. A new Shaft-Key-Connection (SKC) was designed to allow full shaft removal from the driver through a quick-release system operated by a single finger. Figure 4A shows a schematic representation of the SKCs. Key B_1_ in the pressed-down shaft-release-state allows shaft A_1_ to freely move out, while key B_2_ in the default locking state interlocks with A_2_. Each key engages their shaft in their own chassis (C). The keys have an arched shape such that the centre is large enough to allow the instrument shaft to fit entirely through, but the narrower space between the legs fits only around a notch in the shaft. Therefore, once the keys are no longer pressed down they lock the rotation and translation of the shaft with the chassis which have a geared exterior interlocking with the rest of the IM. The spring-loaded keys are easily unlocked by pressing them down, and have self-aligning features when engaging. Additional features of the keys can also force certain orientations of the shafts during attachment, diminishing erroneous assemblies. As in Lenssen et al. (2023b), the end-effector shaft is held by a collet that is manually tightened.

### 2.3 Power coupling and position control

To facilitate the (dis)assembly of the sub-systems a mechanical coupling was designed to automatically align, connect on contact and resume power transfer. The SBI contains a female Hex-Key (HK) (Figure 4B) for each motor output, while the MU and IM carry a male HK at the end of their driver-axes. During coupling of the sub-systems the male keys will use their hexagon shape to fit in the female keys, for which a maximal relative rotation of 30° is required before alignment. Once engaged, the HKs interlock so that torque can be transferred without play, while axial displacement is still possible. The male keys are furthermore spring-loaded to allow full sub-system assembly even if the keys don’t entirely fit.

Spring-loaded brass headers (827-22-004-10-001101, Mill-Max) were used between the sub-systems able to transfer electric power between the MU and the IM, the latter fitted with position feedback sensors (see Section 2.5), while the SBI used their female counterpart. Since the HKs lack an absolute orientation, the position feedback sensors are placed in the IM where they have an absolute relation to the shaft orientation, while their proximity to the instrument can help reduce any backlash effect of the gears and couplings.

During coupling of the IM, immediate contact with the position feedback sensors is made. In an automatic alignment protocol, each motor can then rotate until all HKs have snapped in place, feedback of which is given by the position sensors.

### 2.4 Production

The MU and IM are made almost entirely from aluminium with exception of the collet and SKCs where stainless steel was used. As a cost-effective strategy (Alsoufi, 2017), many parts were water-jet cut from a 4 mm aluminium plate, including the gears required inside the IM. Conventional and CNC machining was able to produce most other parts such as the driver-axes, HKs, SKCs and collet. Solid PEEK bushings were machined to support axial rotation of the driver-axes and female HKs while minimizing part complexity with a focus on cleanability of the IM and SBI in accordance with Table 1. The MU, IM and sensor-house outer casing as well as the SBI were printed from PLA using FDM additive manufacturing, in addition to a cable tunnel inside the IM. Though PLA is not sterilizable, it was chosen as an available interim material for prototyping, keeping the future possibility of injection molding other materials in mind. Finally, extra small stepper motors (GM12-15BY, TT Motors) were used in the MU with local gearboxes with ratio 1:99.

### 2.5 Position feedback

Position feedback is given by an array of four AS5600 contactless angle encoders (manufacturer: AMS) that read the magnetic orientation of permanent neodymium magnets positioned at the end of each driver-axes in the IM. Each sensor can be accessed through an I^2^C protocol which requires 4 connection-lines through a TCA9548A multiplexer (manufacturer: Texas Instruments) between the IM and MU. This enables each sensor to be accessed individually to read out the absolute position of the IM axes, and in extend the instrument.

Sterilization of neodymium magnets is possible regardless the high temperatures required which will affect the magnetic field strengths. The permanent loss is a considerable factor, yet this occurs only once at a first thermal exposure (Trout, 2001). However, since the magnetic field orientation is not affected, neither is the effectiveness of the encoders. Effects of environmental magnetic fields are detected by all sensors equally, which allows this effect to be filtered out.

### 2.6 Mechanical testing

After production and assembly, mechanical testing was done through an identification of play, slack and fitting. Furthermore, the design goals as in Table 1 were validated. The quick-release click fingers of the SBI, as well as in the instrument coupling, were tested through repeated assembly and dis-assembly. General control of the IM’s DOFs, followed by control of the instrument’s DOFs was also assessed. Position sensor feedback was tested through steady-state drift detection as well as position recurrence.

Besides assembly time, having the system operation ready also depends on mechanical interlocking of the sub-assemblies. An experiment was done to test the HKs latching time after dis- and reassembly of the sub-systems. First, the coupled IM was rotated and set to a random orientation after which it was decoupled and extracted. The same was done with the SBI, followed by a final random orientation of the motors of the MU. Then, the SBI and IM were reassembled so that the random axis orientation of each sub-system met at the HKs. Last, all motors were set to rotate until all HKs had latched in place, after which they were stopped. The total time required for each motor to latch was recorded in seconds. This was repeated 10 times. A latch is considered successful if the full axis latches within 2 s as per Table 1.

### 2.7 User testing

To further test the modular design of the sub-systems in accordance to Table 1, a user experiment was set-up to assess the ease of repeated dis- and reassembly. Ten subjects (PhD candidates and master students, Biomedical Engineering department, Delft University of Technology) were included. The study was reviewed and approved by the Human Research Ethics Committee TU Delft. All participants gave informed consent before participation. Given a fully assembled system with an instrument installed, the subjects were shown an instruction video indicating how to dis- and reassemble the device. During the trial a conductor was present to give instructions. The task was as follows: 1) disassembly of the IM with instrument attached, 2) disassembly of the SBI, 3) extraction of the instrument from the IM, followed by the same steps in reverse for a total of 6 steps. For each step the required time was noted. Each component had to be placed down fully between steps. Each participant completed 7 repetitions of a full dis- and reassembly cycle. For this experiment the MU was bolted in a static position while the motors were unpowered.

With relation to the dis- and reassembly method as described in Section 2.1, secondary design requirements were set concerning the required time for each step. Though complete build-up requires the assembly of the IM and instrument, the system can be assembled to a stand-by status with just the SBI, for which the dis- and reassembly time requirements were set as 10 s each, though this does not include the drape. Instrument (IN) insertion and decoupling, which enables inter-operative exchanges, should be done within 20 s for assembly, and 10 for disassembly. Last, IM exchanges facilitating different instruments should be done within 10 s for dis- and reassembly. These secondary requirements have been summarized in Table 2.

**TABLE 2:** Dis- and reassembly time requirements for each sub-system.

Sub-assembly	Assembly	Disassembly
IM	10 s	10 s
SBI	10 s	10 s
IN	20 s	10 s

## 3 Results

### 3.1 System and sub-system design and prototype evaluation

**FIGURE 5:**
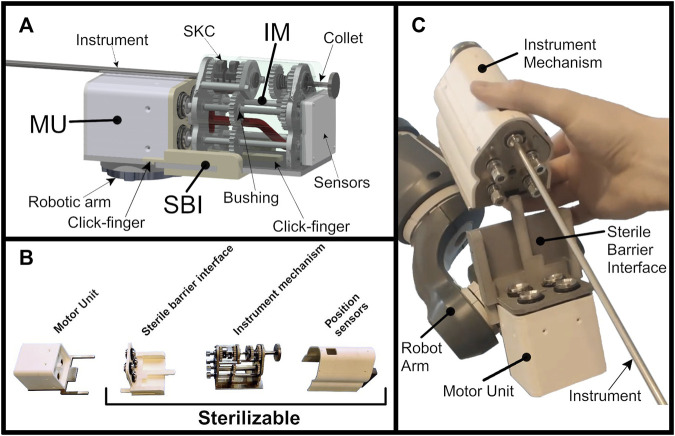
**(A)** The assembled MISLI-Drive with its parts annotated **(B)** The MISLI-Drive separated in its sub-systems **(C)** The MISLI-Drive with separated IM containing the instrument.

All three sub-systems were successfully produced as functional prototypes. Figure 5C shows the MU mounted on an off-the-shelve 7-DOF YuMi IRB 14050 robotic arm (ABB Robotics). Figure 5A furthermore shows a schematic of the driver with its parts annotated. The SBI is attached to the MU through two simple click-fingers, ready to receive the IM which already has the instrument inserted. The backwards facing MU ensures that the IM can be uncoupled away from the patient at any time, when necessary, with the instrument still inserted.

The SBI acts as a divider between the potentially contaminated IM and the clean MU. The SBI is designed to be washable and sterilizable as it is made of hard plastic. The HKs are used to couple the torque of the motors to the IM axes and are supported by PEEK bushings that don’t require moving parts. The axes themselves are secured using o-rings to prevent any contamination from crossing. Similarly, the IM is made of hard plastic and metals, and uses PEEK bushings as guidance.

The size of the total system assembled is 19 cm in length with a total height and width of 8.5 cm. Disassembled, the MU alone is 11 cm × 6 cm × 6 cm. The IM alone is 10.5 cm × 8 cm × 7 cm. The total system weighs 550 g whereas the IM alone weighs 350 g.

### 3.2 Functional testing

After production, the prototype has been tested for all functionalities. A demonstration video (See Supplementary Video S1) shows the fully assembled device mounted on a robot arm. Manipulation of the instrument through control of the motors is visible throughout the video. The quick-release click-fingers for both the SBI and the IM allow detachment of both as stated in the design requirements in Table 1. Backwards IM detachment can be done with a single hand. Instrument removal and reinsertion is also shown, along with an example of instrument tip-exchange while the instrument shafts are still coupled. Dis- and reassembly times are discussed in Section 3.5. After assembly of the SBI and IM into the pre-attached MU, control of the instrument is near instantaneous, as further tested in Section 3.4.

The stepper-motors have a resolution of 0.182° which translates directly to tip and shaft rotation, though gear backlash was a factor. Articulation reaches a control accuracy of 0.152° through the SATA joint. The position feedback sensors were able to measure the orientation of all four axes in the IM. Through a 12-bit I^2^C output, the AS5600 sensors are accurate to about 0.088° of rotation for the full 360° range, which translates to 0.07° articulation through the SATA joint, which helps to compensate control inaccuracies. Experimental testing showed the instrument’s level of accuracy to succeed practical requirements. Boot-up and re-connection of the sensors is near instantaneous after IM assembly. Master-slave control latency is about 25 ms. The sensors are easily removed from the back of the IM for inspection and cleaning, though all components involved in the sensing strategy are sterilizable. Due the contactless nature of the sensor-axis relation, no moving parts are required, while the sensors can be shielded from direct contact with the inside of the IM.

### 3.3 Modular build

Instrument shaft insertion is done simply through pressing down the motor-aligned SKCs for which a finger-size window is available at the top. Further attachment of instrument end-effectors is done through the use of the collet at the back of the IM. Instrument insertion into the IM can be done while the IM is engaged with the system or separately on a table-top. Instalment of the SBI is done once before operation and is removed only once after.

After use, the SBI and IM are detached. The further detachment of the IM cover, which includes the position sensors, is possible although all included electronics are also sterilizable. Removal of the IM cover exposes the internal axes, gears and bushings for specific cleaning and inspection (see Figure 5B), which was a specific design requirement as mentioned in Table 1.

### 3.4 Hex-latching experiment

**FIGURE 6:**
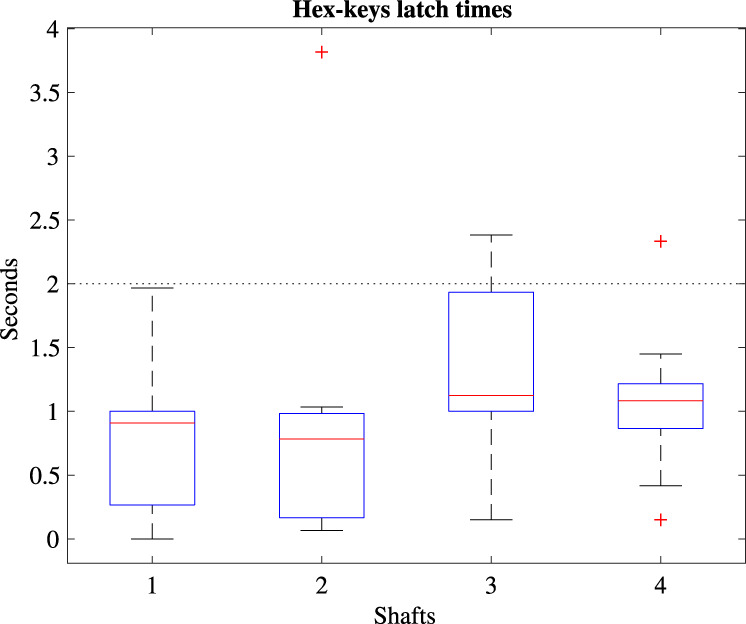
Boxplots of the time required to latch for each axis with a dotted line indicating the time requirement.

Figure 6 shows the time required for each axis to fully latch using the data of 10 repetitions, making a total of 80 HK latches. The figure shows the individual axes shared between the three sub-systems, each containing two HKs. A dotted line is added to indicate the time goal of 2 s. A median of 0.98 (0.07–2.38) (min-max) seconds is required for all axes to fully latch. 97% of the axes latched within 2 s, while 8/10 times all axes fully latched within 2 s, with a maximum recorded axis outlier of 3.8 s. A video clip showing all 10 repetitions is also available in Supplementary Video S2.

### 3.5 Dis- and reassembly experiment

**FIGURE 7:**
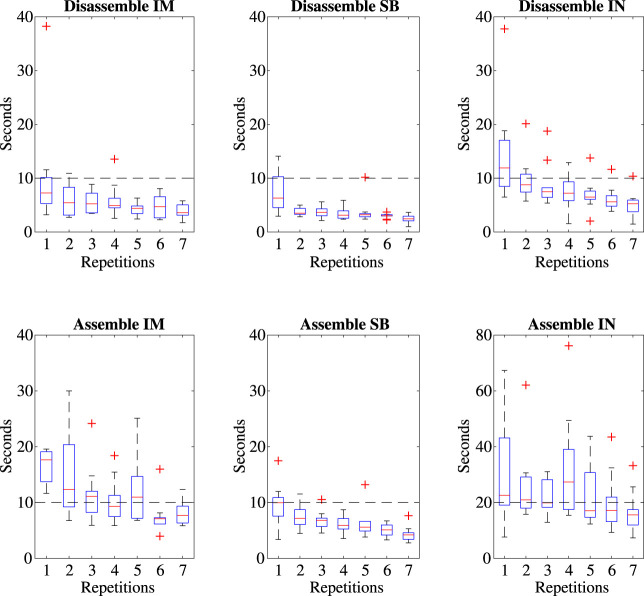
Boxplots of the time required for each part of the dis- and reassembly of the device including lines indicating the design goals (Table 2).

Figure 7 shows the boxplots for each step in the dis- and reassembly cycle for the 7 repetitions, excluding subject 3 which caused 54% of the outliers. Notice that the bottom row was done in reversed order. Horizontal lines are added to each plot to express the design requirement time goal as per Table 2. As an indication of the participants final proficiency, the median and range of the last two repetitions have been taken. For disassembly of the IM, SBI and IN followed by assembly of the IN, SBI and IM, the median time (and range) required was 3.7 (1.8–8.1), 2.8 (1.0–3.7), 5.3 (3.2–7.8), 15.2 (7.4–25.6), 4.4 (2.8–6.4) and 7.0 (4.0–9.6) seconds respectively.

**FIGURE 8:**
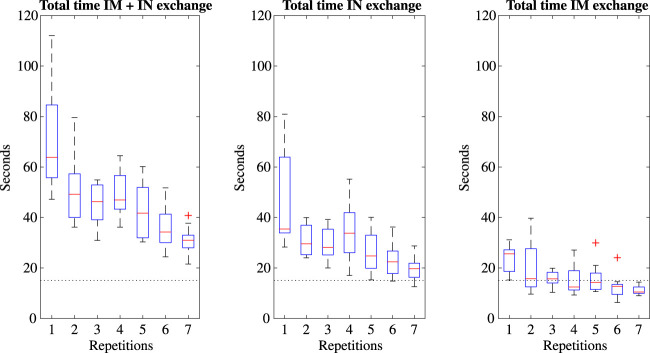
Boxplots of the time required for different methods of instrument exchange, including a dotted line indicating the time requirements.

For an instrument exchange during operation, removal of the SBI and IM is unnecessary. We can therefore combine the time requirement of IN dis- and reassembly for instrument exchange only. Similarly, it is possible to have a second IM with a different instrument already attached waiting for an IM exchange as a means to switch instruments. Figure 8 shows the boxplots of the time required when combining IM and IN exchange. Taking the final two repetitions as representatives, it shows that IN exchange is faster than IN exchange after IM decoupling, yet that IM exchange with a potential second, differently equipped IM is faster still. Respectively, exchange times were found as 31.5 (21.6–51.7), 21.3 (12.6–36.3) and 11.0 (6.3–14.6) seconds. IM exchange therefore meets the instrument exchange time requirement as set in Table 1. Datasets are available at Lenssen et al. (2023a).

## 4 Discussion

This work has shown that the MISLI-Drive is able to hold the SATA-LRS and control each of its 4 DOFs. The design of the driver fulfills the set of specific focus points as stated in Table 1. The MISLI-Drive makes inter-operative instrument exchanges possible and has a general modular build for part exchange. Cleanability, another requirement, led to the dis-attachment of the IM for sterilization and inspection. General cost-reduction was designed through the use of simple production methods, exchangeable parts during maintenance, and a general re-use of the system. Position feedback sensors have refined the control of the instrument which can now have multiple forms through the standardized SKC clutching mechanism. The following sections will discuss in detail the design requirements and their acquired results, as summarized in Table 3.

**TABLE 3:** An overview of the design requirements and their results.

#	Design requirements	Goal	Result [median (range)]
1	Instrument is detachable from driver	All shafts and end-effector	All shafts and end-effector are detachable
2	MI is detachable from MU	Remove IM from the sterile site	IM is removable and separated by the SBI
3	Adaptive instrument clutching	Shaft size of 3–5 mm	The SKC’s can clutch 7 mm diameter and 5 mm instruments
4	Low instrument exchange time	15 s	IM exchange possible in 11.0 (6.3–14.6) seconds
5	No arm reorientation required	Independent from arm	IM exchange is independent from arm
6	Instant instrument control	2 s	IM and sensors operational in 0.98 (0.07–2.38) seconds
7	Precise instrument control	0.5° accuracy and 50 ms latency	0.152° articulation and 25 ms latency
8	Focus on cost reduction	Production price of 200€	Efficient design cost less than 200€ to produce
9	Reusable system through cleanability	Internal surfaces exposable	All internal surfaces of IN, IM and SBI are exposable
10	Low weight	500 g or less	Total system at 550 g, IM at 350 g

### 4.1 Instrument shaft connection

The SKC mechanisms have proven to be intuitive and effective as a quick-release system for the instrument shafts. The advantage of complete shaft removal allows for complete shaft disassembly which improves cleanability and inspection of both the instrument and the IM. The SKCs have a standardized clutching mechanism able to hold and control any shaft that fits a simple notch. The current SKC design is able to fit diameters of 7 mm or lower, where the SATA-LRS is a 5 mm instrument, though this mechanism could be scaled up to include 12 mm shafts as well.

### 4.2 Instrument exchanges

In spite of the benefits of the SKC design, instrument exchange time is slightly slower at 5.3 (3.2–7.8) seconds for disassembly and 15.2 (7.4–25.6) seconds for reassembly compared to the original SATA-Drive’s PPC clutch at 2.5 (0.5–4.0) and 6.0 (4.0–12.0) seconds for the same tasks. However, as discussed in Section 3.5, IM exchange is comparable to the PPC at 3.7 (1.8–8.1) and 7.0 (4.0–9.6) seconds for dis- and reassembly. The two methods of instrument exchange have a focus on different aspects of the driver’s use. Preparing a secondary IM with an alternative instrument pre-assembled allows for quick, inter-operative instrument exchanges through replacement of the IM, while immediate exchanges of the instrument alone reduces costs in procurement, cleaning and personnel. IM exchange also allows for a full backwards retraction, requiring no reorientation of the robotic arm, whereas direct instrument exchange does. The instrument exchange time goal set in Table 1 was decided compared to the Da Vinci system whose instruments come with a mechanism similar to the IM permanently attached (Inuitive, 2021). Sgarbura and Vasilescu (2010) had trained personnel perform an average instrument exchange at 7.7 (3.7SD) seconds, matched with the untrained participants of this work. The method of IM exchange is slightly slower but comparable at a median of 11 (6.3–14.6) seconds, and thereby meets our goal of 15 s. Instrument exchange without changing the IM is also a time-feasible option. An additional advantage of IM switches could be the modular capacity of installing different IM’s adapted to alternative instruments not based on the SATA technology, or with different electrical or mechanical requirements, each of which would require the procurement of only one IM. Where the MU stays semi-permanently attached to the robot arm, the IM and instrument weigh 350 g, about 100 g more than an average 5 mm Da Vinci grasper (Inuitive, 2021), though these adopt plastic casings and parts. A weight reduction of the MISLI-Drive could be pursued in the future by replacing metal parts for similar hard plastics.

### 4.3 Cleanability, modularity and re-use

The preliminary use of PLA as a prototyping plastic should in the future be replaced for hard plastics fit for sterilization and injection molding such as PP, PSU or PEEK (McKeen, 2014).

The choice of a detachable IM comes with a slight increase in handling complexity, yet noticeably offers better feature access for cleanability and inspection, feasibly improving residual contamination. While the Da Vinci instruments with their permanent version of the IM rely fully on blind flushing, the IM is designed for disassembled cleaning, inspection and maintenance. It is this focus on cleanability and inspection that promotes this design’s continuous re-use, rather than limited re-use. This is further supported by the modular ability to inter-exchange parts during use or maintenance rather than replacing the whole system, which allows minimal adaptation to new circumstances. It is expected that repair on component level, as well as re-use of the driver and instrument though perpetual cleaning, removes a financial burden in procurement, reducing the overall cost of surgery (Rizan and Bhutta, 2022). General re-use, reprocessing and remanufacturing of surgical systems has the potential to significantly reduce surgical waste, making this system more sustainable as per the Circular Economy philosophy (van Straten et al., 2021). Notably, maintenance and re-use are seen as the most effective cycles to keep materials in the economic circle, and require the least energy, therefore being the best options for the environment (MacArthur, 2013).

### 4.4 Initialization and resumed use

The use of the orientation encoders allows for instantaneous recognition and control of the IM through the absolute rotation feedback of the shafts. This plug-and-play feature can optimize HK latching and protect the installed instrument from over-articulation. At first contact the driver can instantly recognize the orientation of the shafts due the absolute fit of the SKCs. Use of the shaft rotation feedback can also diminish slack and backlash of the gears used in the IM, combined with pre-emptive stepping as described in Lenssen et al. (2023b).

Resuming work immediately after assembly supports the mid-procedure instrument exchanges requiring no reorientation rather than the need for pre-equipped stand-by robotic arms. Reducing the number of active robotic arms could lower surgical costs, thereby making robotic surgery more affordable and accessible. Secondly, fewer deployed robotics arms around the patient could greatly improve access to the surgical site which is sometimes difficult for surgical assistants Nezhat et al. (2006).

### 4.5 Comparison to other systems

The Da Vinci system has earlier been referenced for their fast instrument exchange times and their market position. Their permanent instrument gearboxes have a similar quick-release system as the IM presented in this work, yet, their internal cable structures makes the system unfit for post-cleaning inspection which has limited their life-cycle. The company ‘Medtronic’ has developed the Hugo RAS system which uses comparable gearboxes to connect cable-driven steerable, single use instruments (Medtronic, 2023). The company “Asensus” has similarly developed a set of modularly applicable, cableless, reuasble instruments, with attached gearbox, though these are not articulated. Their steerable instruments are disposables (Darwich and Stephan, 2022). The company “Distalmotion” has had a different interest in instrument modularity and instrument exchange. The instruments are without a gearbox and are easily dis- and reassembled by insertion through the robot arm which requires shielding similar to the SBI as presented in this work. Though their instruments have a significantly reduced complexity making them more sustainable, they are not reusable (Digitalmotion, 2023).

Elements of plug-and-play instrument exchange, reduced instrument complexity and reusable systems are thus features of interest on the current market. However, as the state of the art competes with unique aspects, no system currently includes all aspects. The MISLI-Drive promises to be a fully reusable, cableless, modular system with a dynamic and sustainable use for fully articulated instruments, which is a unique combination.

### 4.6 Limitations

This work has been done as part of an early development prototype phase. As such, although the system proves to be functional, the influence of wear and operation time on the sensitivity and accuracy of the sensors and components is yet to be measured in a final version of the design. Similarly, the use of a hard plastic for the SBI and MU and IM covers is yet to prove practical compatibility with recurring sterilisation methods. Finally, a Live Cycle Assessment and Health Technology Assessment are needed to truly prove the costs and sustainability benefits of this mechanism above alternatives.

## 5 Conclusion

The MISLI-Drive has been successfully designed to manipulate the reusable and steerable SATA-instruments. Focusing on sterilizability, the driver has been designed to be fully reusable rather than the limited re-use as seen on the market. Through a modular approach, the system’s sub-assemblies can be replaced or repaired when necessary. These modular features are expected to reduce the need for part purchasing costs which is further enabled through the continuous re-use of the system as a whole. Also in the focus on the Circular Economy philosophy and the footprint on the environment, these developments are expected to bring positive outcomes, hopefully shifting the tendencies of other devices on the surgical market.

Two different methods exists for instrument exchanges focusing on speed and cost-effectiveness, both possible within seconds. The backwards facing MU ensures decoupling away from the surgical site which could support instrument exchanges mid-procedure without robot reorientation. The use of the HKs combined with the absolute rotation encoders have shown the device to have plug-and-play characteristics when re-assembling the modules. These quick-release features enable a more versatile, adaptive and sustainable use of components in the field of robot surgery, and are compatible with a range of SATA-instruments in a standardized fashion. The stand-alone driver is applicable to high level robotic arms fit for a fully utilised operation room, but due to its low cost, is also available for simple set-ups in more rural environments. Beyond surgical settings, the modularity and cleanability features could also be valuable in robotic systems dealing with chemical or biological contamination that require cleaning of the manipulator. Overall, the MISLI-Drive is a step towards more accessible and modular robotic surgery.

## Data Availability

The datasets presented in this study can be found in online repositories. The names of the repository/repositories and accession number(s) can be found below: DOI: 10.4121/72aeb227-d5f3-431b-9b82-f5219c47d05c.
